# Metabolite Changes of *Perna canaliculus* Following a Laboratory Marine Heatwave Exposure: Insights from Metabolomic Analyses

**DOI:** 10.3390/metabo13070815

**Published:** 2023-07-03

**Authors:** Awanis Azizan, Leonie Venter, Peet J. Jansen van Rensburg, Jessica A. Ericson, Norman L. C. Ragg, Andrea C. Alfaro

**Affiliations:** 1 Aquaculture Biotechnology Research Group, School of Science, Auckland University of Technology, Private Bag 92006, Auckland 1142, New Zealand; awanis.azizan@aut.ac.nz (A.A.); leonie.venter@aut.ac.nz (L.V.); 2 Human Metabolomics, North-West University, Potchefstroom 2520, South Africa; peet.jansenvanrensburg@nwu.ac.za; 3 Cawthron Institute, Private Bag 2, Nelson 7042, New Zealandnorman.ragg@cawthron.org.nz (N.L.C.R.)

**Keywords:** aquaculture, marine heatwave, metabolomics, mussels, New Zealand, *Perna canaliculus*, pathways, temperature, stress

## Abstract

Temperature is considered to be a major abiotic factor influencing aquatic life. Marine heatwaves are emerging as threats to sustainable shellfish aquaculture, affecting the farming of New Zealand’s green-lipped mussel [*Perna canaliculus* (Gmelin, 1791)]. In this study, *P. canaliculus* were gradually exposed to high-temperature stress, mimicking a five-day marine heatwave event, to better understand the effects of heat stress on the metabolome of mussels. Following liquid chromatography-tandem mass spectrometry analyses of haemolymph samples, key sugar-based metabolites supported energy production via the glycolysis pathway and TCA cycle by 24 h and 48 h of heat stress. Anaerobic metabolism also fulfilled the role of energy production. Antioxidant molecules acted within thermally stressed mussels to mitigate oxidative stress. Purine metabolism supported tissue protection and energy replenishment. Pyrimidine metabolism supported the protection of nucleic acids and protein synthesis. Amino acids ensured balanced intracellular osmolality at 24 h and ammonia detoxification at 48 h. Altogether, this work provides evidence that *P. canaliculus* has the potential to adapt to heat stress up to 24 °C by regulating its energy metabolism, balancing nucleotide production, and implementing oxidative stress mechanisms over time. The data reported herein can also be used to evaluate the risks of heatwaves and improve mitigation strategies for aquaculture.

## 1. Introduction

An organism’s optimal physiological functioning occurs within certain temperature limits, determined by thermal conditions experienced over evolutionary timescales. Beyond these limits, physiological function declines, and without mitigating action, mortality occurs [1]. In New Zealand (NZ), seawater temperatures are currently reported as several degrees above normal conditions, with more frequent and severe events projected [2]. In line with these projections, extreme events are likely to have an impact on the rich and diverse marine ecosystems, aquaculture facilities, and commercial and recreational fishing grounds found around NZ [3]. In particular, elevated summer seawater temperatures and marine heatwaves (MHW) are causing mortalities in farmed green-lipped mussels (*Perna canaliculus*) [4]. Considering that mussel farming is the cornerstone of the NZ aquaculture industry [5] and that the industry aims to grow significantly over the coming decade [6], data are needed to support efforts in safeguarding aquaculture production against marine heatwaves.

In broad terms, MHW are defined as periods where temperatures exceed the 90th percentile of the local climatology for five days or more [1]. It is believed that MHW could have greater ecosystem and evolutionary impacts within marine systems than the more gradual effects of climate change. Here, heatwaves are said to compound the effects of underlying warming trends with limited opportunity for organisms to adapt or acclimate, while the slower effects of warming allow more time to process the change with adaption or acclimation to follow [7].

Laboratory-based thermal studies on *P. canaliculus* support the use of 16 °C as a control condition, with mortalities starting to occur when seawater temperatures reach 24 °C [4]. Changes in sea surface temperatures affect marine organisms at all levels of biological organisation, including behavioural, molecular, biochemical, and physiological responses [8]. Typically, the metabolic response to thermal stress includes an increase in energy production to maintain homeostasis, utilising various metabolic pathways and oxidation of multiple energy substances, such as proteins and lipids [9]. In *P. canaliculus*, metabolomics has been used to create a blueprint of metabolite pathways affected in response to thermal stress. Some of the key responses are (1) increases in tricarboxylic acid (TCA) cycle intermediates and metabolites feeding into the TCA cycle to sustain energy production via oxidative phosphorylation, (2) the activation of anaerobic metabolic pathways to support rapid energy production, (3) the use of substrate-level phosphorylation enabling the synthesis of adenosine triphosphate (ATP) directly from the phosphorylation of adenosine diphosphate (ADP), and the utilisation of the fatty acid oxidation to supply ATP molecules [4,10,11]. What has not been established is the short-term responses implemented by mussels at the metabolite level during a MHW event. Insight into the physiological response of mussels to a stressor can be utilised to understand their resilience in a changing environment. Additionally, new information relating to mussel responses to stressors can be used to improve farming and processing efficiency, which adds value to the aquaculture industry [12]. Data representative of a response at a specific time can be obtained by utilising metabolomics approaches, targeting the phenotype of an organism, and expanding on what is happening on a metabolic and physiological level [13]. Metabolomic analysis hold the promise to simultaneously monitor precursors, intermediates, and products of metabolic pathways, acting as a discovery tool to detect metabolites affected by stressors [14].

Considering all the above, the objective of this study is to investigate the metabolic changes that occur in *P. canaliculus* (green-lipped mussel) when exposed to a laboratory-based MHW for a period of five days. By gaining a comprehensive overview of these metabolic changes, the study aims to enhance our understanding of the physiological responses of *P. canaliculus* to heat stress and its implications for overall mussel health and survival. We hypothesise that the metabolic changes observed will reflect adaptations to heat stress, including shifts in energy metabolism and alterations in key metabolic pathways. Moreover, the data reported herein can be used to support aquaculture industry initiatives and policies to evaluate the risks of MHW and improve mitigation strategies.

## 2. Materials and Methods

### 2.1. Animal Husbandry and Haemolymph Collection

Adult *Perna canaliculus* (Gmelin, 1791) (n = 150) were collected from a commercial farm (SPATnz) located within the Marlborough Sounds, New Zealand, and transported to Cawthron Institute’s Te Wero facility in Nelson, New Zealand. Upon arrival, the mussels were placed in a flow-through seawater system at ambient temperatures (±16.04 °C, AVE ± STDEV over acclimation period). Mussels were held for six days to acclimate to holding conditions and fed *Tisochrysis lutea*. Water flow rates were kept at 2–3 L/min and aerated with air-stones. Seawater temperature, ammonia, nitrite, and nitrate levels were monitored daily.

**Figure 1:**
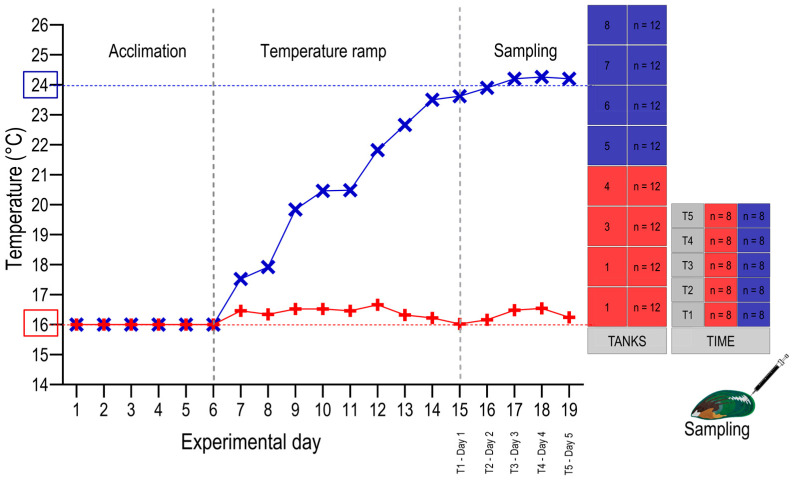
During the marine heatwave exposure, mussels were divided into eight tanks, with the first half of the tanks kept at ambient temperature of 16 °C (red line) and the second half of the tanks experiencing a moderate temperature increase over time, resulting in a target temperature of 24 °C (blue line), whereafter haemolymph sampling was carried out for five days at the target temperatures.

For experimental purposes, 96 animals were divided into eight tanks (i.e., 12 animals per tank). Mussels in tanks one to four were kept at ambient conditions of 16 °C, serving as the control temperature for this experiment (correlating to mean temperatures experienced in the wild in the summer in the Marlborough Sounds). Mussels within tanks five to eight were slowly exposed to an increase in seawater temperature of 1 °C per day until 24 °C was achieved, whereafter, the set condition at 24 °C was maintained for the duration of the experiment. Temperatures were achieved via a heat exchanger connected to a hot/cold loop, controlled by solenoid valves (Vaportec LTD, Napier, New Zealand), and monitored daily (Figure 1). Once experimental temperatures were achieved, sampling started.

At days 1, 2, 3, 4, and 5 (24, 48, 72, 96, 120 h post-exposure, respectively) of the experiment, haemolymph was collected from two animals per tank, per condition (resulting in 8 mussels per timepoint, per temperature). Prior to haemolymph sampling, the animals were patted dry with paper towels and weighed to the nearest 0.01 g, and the shell lengths were measured to the nearest 0.10 mm along the longest axis, using callipers. The animals were gently opened on the ventral posterior side to access the posterior adductor muscle, as previously described by Ericson et al. (2023). Using a pre-chilled 23-gauge needle attached to a 1 mL sterile syringe, haemolymph was collected and placed into a microcentrifuge tube. A volume of 400 µL (with 20 µL of 10 mM L-alanine-2,3,3,3-d_4_ internal standard) was transferred to a microcentrifuge tube and snap-frozen using liquid nitrogen and stored at −80 °C until subsequent use [4]. Additionally, mussel gender was assessed by observing the colour of the gonad, which is a cream (milky white) colour in males and a yellow (orange) colour in females, with 33 females and 47 males recorded.

### 2.2. Metabolomics Sample Preparation, Analysis, and Data Processing

Frozen haemolymph samples were dried under vacuum for 4 h at 0 °C. A two-step sequential extraction method was used for metabolite extraction. In brief, a volume of 500 µL cold methanol:water solution (50% MeOH:50% H_2_O) was added to the dried sample and vortexed for 1 min, followed by centrifugation at 3500 rpm for 5 min at −9 °C. The supernatant was collected and transferred to a new tube, whereafter, the remaining sample was re-extracted by adding 500 µL cold methanol:water solution (80% MeOH:20% H_2_O), vortexed and centrifuged (as above). Supernatants were collected from the re-extracted sample and pooled with the first collection, placed back in the −80 °C freezer overnight, and dried in a SpeedVact concentrator [15]. A volume of 200 µL 50% MeOH:50% H_2_O was added to the dried sample, vortexed, and centrifuged (10 min, 10,000 rpm), whereafter, 100 µL was transferred to a vial containing a pulled point glass insert for liquid chromatography-tandem mass spectrometry (LC-MS/MS) analyses.

Quality control (QC) samples were included within the biological sample batches and prepared by taking a 50 µL of each biological sample, thoroughly mixed into a homogenous pooled sample [16] and treating them as experimental samples. The QC samples were injected at regular intervals throughout the analytical run of the analysed batches to measure repeatability and identify any potential batch effects in the data.

Metabolomics analyses were conducted by running samples on an Agilent 1260 LC coupled to an Agilent 6470 triple quadrupole (QQQ) mass spectrometer (Agilent Technologies, Santa Clara, CA, USA). Agilent MassHunter Workstation Data Acquisition (Version 10.0) was used for compound calibration and data acquisition. For all analyses, the column was kept at 40 °C, and 2 μL of sample was injected. The flow rate was kept at 0.25 mL/min. For ion-pairing chromatographic separation, an Agilent ZORBAX Extend C18 column was used with the mobile phases prepared as follow: Mobile phase A: 97% water, 3% methanol, 10 mM tributylamine, 15 mM acetic acid, and 5 μM medronic acid (Agilent infinity lab deactivator additive). Mobile phase B: 10 mM tributylamine, 15 mM glacial acetic acid, and 5 μM medronic acid. Channel A and C delivered mobile phase A and B, respectively, while channel B contained isopropanol, and channel D delivered acetonitrile to wash the column at the end of the run. The following gradient was used: 0–2.5 min, 100% A at 0.25 mL/min; at 7.5 min, 80% A; at 13.0 min, 55% A; at 20.0 min, 1% A and kept till 24.0 min. From 24.1–27.0 min, the 99% C was changed to 99% D. From 27.1–31.4 min, the flow rate with 1% A and 99% D was increased to 0.8 mL/min flow rate; from 32.3–40.0 min, the flow rate was steadily decreased and switched back to 100% A and reduced back to 0.25 mL/min. The mass spectrometer was operated in negative ionisation mode using an Agilent Jetstream ESI source with the following parameters: A gas temperature of 150 °C, Gas flow of 10 L/min, Nebulizer gas pressure of 45 psi, Sheath gas temperature of 325 °C, Sheath gas flow of 12 L/min, Capillary voltage 2000 V, Delta electron multiplier voltage (Delta EMV) 200 V. Dynamic MRM scans were used with 0.07 min peak width and acquisition time of 24 min. Delta retention time of plus and minus 1 min, fragmentor voltage of 40 eV, and cell accelerator voltage of 5 eV were incorporated into the method [17]. Tuning and calibration of the QQQ were achieved with Agilent ESI Low Concentration Tuning Mix.

**Figure 2:**
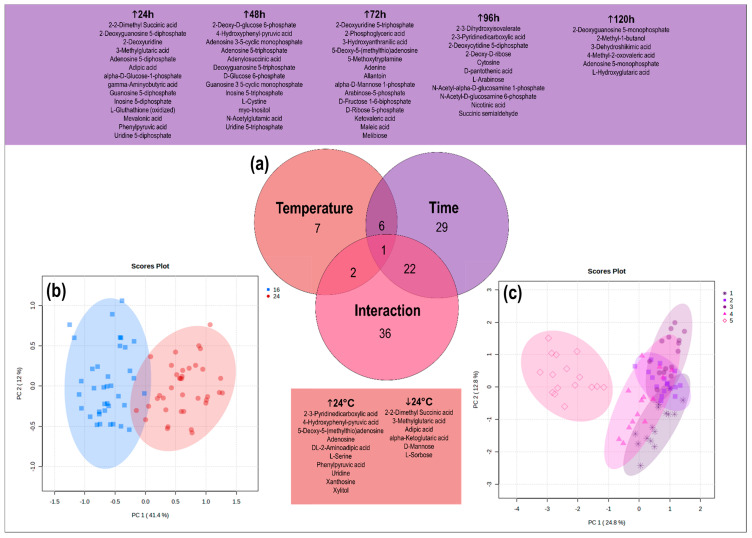
(**a**) Overview of metabolomics results as a Venn diagram focusing on significantly different metabolites due to the effect of temperature, time, and an interaction effect. Additionally, PCA plots are shown of mussels (**b**) exposed to two temperatures (16 and 24 °C) and (**c**) sampled at five timepoints (24, 48, 72, 96, 120 h).

**Figure 3:**
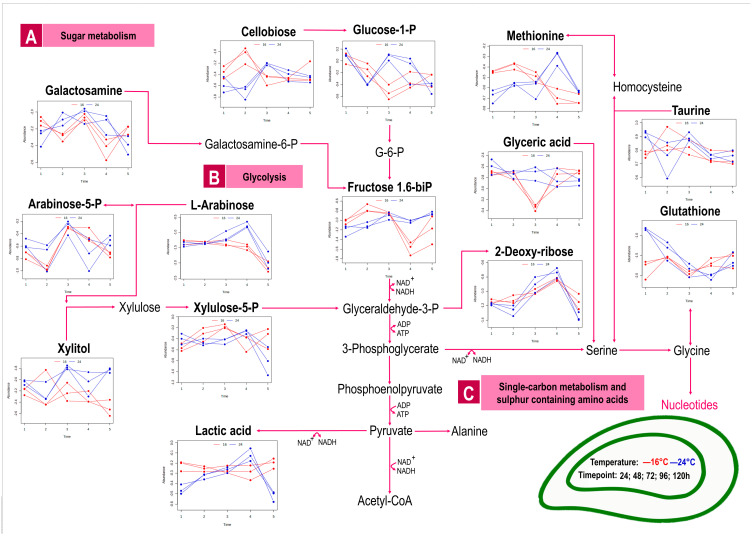

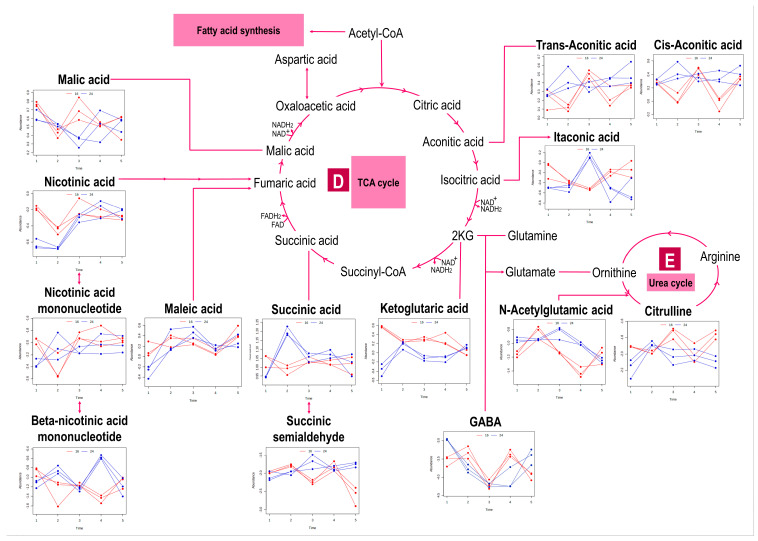

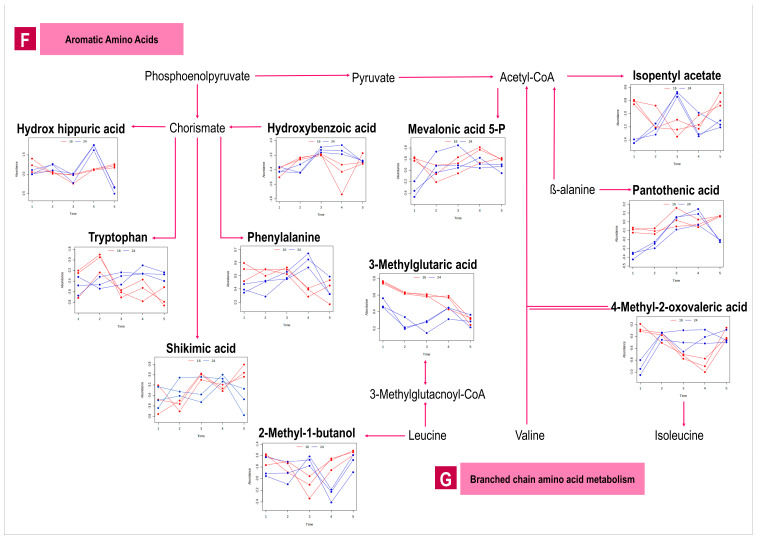

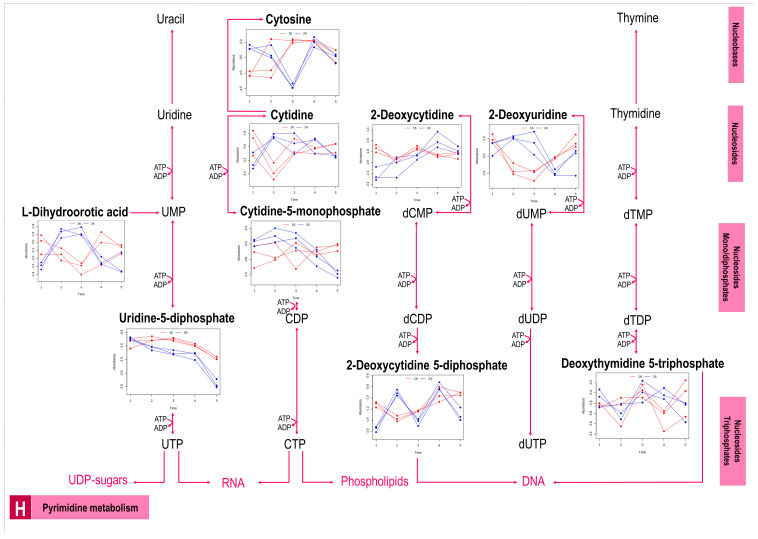

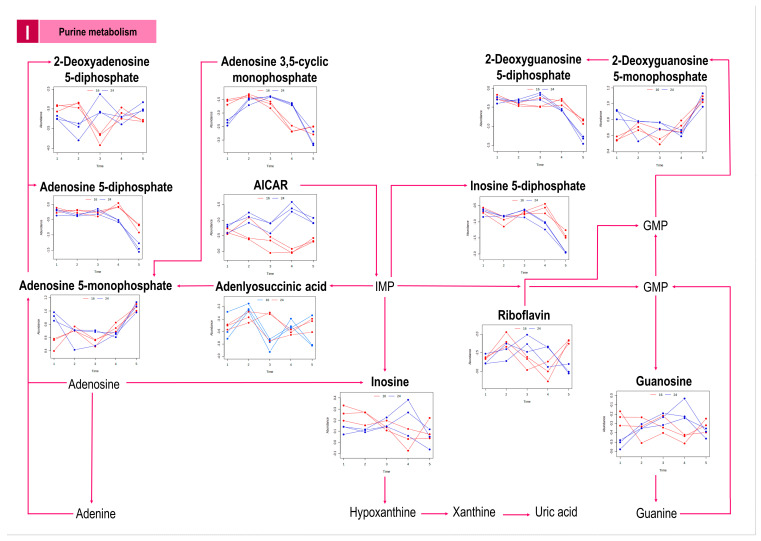
Perturbed metabolites from carbohydrate metabolism (**A**), the glycolysis pathway (**B**), and single carbon metabolism (**C**) detected in haemolymph of *P. canaliculus* following exposure to 16 °C (control) and 24 °C (heat stress) and five sampling timepoints (24, 48, 72, 96, 120 h). Perturbed metabolites connected to the TCA (**D**) and urea cycle (**E**) detected in haemolymph of *P. canaliculus* following exposure to 16 °C (control) and 24 °C (heat stress) and five sampling timepoints (24, 48, 72, 96, 120 h). Perturbed metabolites linked to amino acid (**F**,**G**) metabolism detected in haemolymph of *P. canaliculus* following exposure to 16 °C (control) and 24 °C (heat stress) and five sampling timepoints (24, 48, 72, 96, 120 h). Perturbed metabolites associated with pyrimidine metabolism (**H**) detected in haemolymph of *P. canaliculus* following exposure to 16 °C (control) and 24 °C (heat stress) and five sampling timepoints (24, 48, 72, 96, 120 h). Perturbed metabolites associated with purine metabolism (**I**) detected in haemolymph of *P. canaliculus* following exposure to 16 °C (control) and 24 °C (heat stress) and five sampling timepoints (24, 48, 72, 96, 120 h).

The data were pre-processed with Agilent MassHunter Workstation Quantitative Analysis Software (Version 10.0). Two unique transitions were monitored per individual metabolite to provide spectral matching in addition to retention time, resulting in metabolite identities with the highest level of confidence [18,19]. To remove non-biological variation, the data were normalised using the mass spectrometry total useful signal normalisation method [20]. The data were generalised log transformed to alleviate the dependency of the variance on the compound concentrations [21]. Two-way ANOVA was used to determine the influence of time (days 1–5) and temperature (16 and 24 °C) on the metabolite response of mussels (between subjects, *p* < 0.001). Herein a Venn diagram was generated to summarise the statistically significant changes in metabolite levels associated with each factor, along with their interactions (Figure 2a). The metabolite response pertaining to time and temperature was future analysed and visualised in a blocked manner [22], with principal component analysis (PCA) (Figure 2b,c). Statistically significant metabolites with an interaction effect, as indicated by the metabolite interaction patterns between temperatures (of the control and treatment groups) over time [23], were manually plotted within metabolic maps (Figure 3).

## 3. Results and Discussion

Exposure to environmental stressors like temperature often leads to elevated costs of basal metabolism, with increased energy requirements as the main outcome [24]. This was demonstrated in the present study, where LC-MS/MS data of *P. canaliculus* haemolymph were used to investigate the metabolic processes of mussels exposed to 24 °C compared to the control group of mussels held at 16 °C over a five-day period. The metabolite response was a consequence of mussel exposure to a gradual increase in seawater temperature, which reached a target temperature (24 °C), followed by holding and monitoring at that temperature for 5 days. A total of 182 metabolites were detected, with 16 metabolites affected by temperature, 58 metabolites affected by time, and 61 metabolites showed significance due to an interaction effect between time (T1-5) and temperature (16, 24 °C) (Figure 2a, Table S1). From the PCA score plot representing temperature (Figure 2b), a moderate overlap in scores were seen, showcasing the lower impact of this experimental factor on the metabolism of mussels. The relevant metabolic variation due to temperature is mainly captured by PC1, explaining 41.4% of the variance. The PCA score plot relating to time (Figure 2c) showed the smallest grouping within the first timepoint and a shift in metabolite response as time progresses. Herein timepoint five showed a clear grouping from the other timepoints, depicting an opposite metabolite response. Of importance for discussion in this manuscript is the metabolites relating to an interaction between temperature and time. These metabolites were mapped and interpreted in terms of pathways relating to (A) sugar metabolism; (B) glycolysis; (C) single-carbon metabolism and sulphur containing amino acids; (D) TCA cycle; (E) urea cycle; (F) aromatic amino acids; (G) branched chain amino acid metabolism; (H) pyrimidine metabolism and (I) purine metabolism (Figure 3). In the following sections, we discuss the overtime metabolite response shown by an increase or decrease in metabolite abundance as reflective of the heat-stressed mussels (in comparison to the controls).

### 3.1. Sugar and Carbohydrate Metabolism to Support Energy Storage or Utilisation

Typically, carbohydrates are the main source of energy metabolism and play a vital role in cell homeostasis under stressful conditions [25]. The overall response in the sugar-based metabolites (Figure 3, pathway A) of the heat-stressed mussels under investigation was a decrease in metabolite concentration after 48 h, followed by an increase at 72 h. Metabolites, such as cellobiose, glucose-1-phosphate, fructose-1.6-biphosphate, galactosamine, arabinose-5-phosphate, arabinose, and xylitol, increased after three days at a constant temperature of 24 °C when compared to control temperature 16 °C. It can be argued that mussels experienced an energy deficit by 72 h due to the presence of heat stress and increased these sugar metabolites to support increased glycolysis functioning (Figure 3, pathway B). The conversion of various types of sugar metabolites (glucose-1-P, fructose 1.6-biP, xylitol, xylulose-5-P) as an influx to the glycolysis pathway [26,27] in response to heat stress is herein reported for the first time in *P. canaliculus*. Ultimately glucose flux supported substrate-level phosphorylation in the current study, where ATP was directly phosphorylated from ADP, as previously described in thermally stressed *P. canaliculus* [10]. An increase in glucose was also seen in the haemolymph of *Mytilus edulis* to meet increased tissue energy demands in response to temperature stress [28]. The accumulation of sugars may also help to stabilise proteins against heat stress [29] and support the biosynthesis of defensive compounds induced by stress [30]. Additionally, sugar metabolites are capable of inducing antioxidant activity against oxidative stress [31]. Furthermore, the current study indicates that the sugar-based metabolites entering the top intermediates of the glycolysis pathway had similar metabolite levels at both 16 and 24 °C by day five, suggesting that an equilibrium had been reached between the depletion and utilisation of sugar metabolites in response to thermal stress. The xylitol-based metabolites showed opposite temperature trends by the end of the experiment, with both temperature groups ending with different metabolite concentrations. Both L-arabinose and xylose are used as carbon sources by a variety of microorganisms [32] and plants [33], while the functions of these metabolites have not been characterised in mussels to date.

### 3.2. TCA Cycle as Central Hub for Energy Metabolism and Redox Balance

In the current study, a steady supply of ATP production during thermal stress was further secured via changes within the TCA cycle (Figure 3, pathway D). Affected metabolites, such as itaconic acid, ketoglutaric acid, succinic acid, and malic acid, remained higher in the control group of mussels (16 °C) after 24 h of the experiment. It was only after 48 h that the metabolite concentrations of the temperature-stressed group (24 °C) were similar to the 16 °C group or higher. Increased TCA cycle intermediates were previously found in *P. canaliculus* following a 3 h acute thermal challenge [34] and after 60 min of severe heat shock [10]. Considering that the metabolite pathways preceding the TCA cycle (i.e., glycolysis, pentose-p-pathway) did not show increased metabolites at timepoint one (24 h), an influx of intermediates is not experienced within the TCA cycle from the start. Yet, as time proceeded, the effect of temperature stress was seen as additional metabolite products were used to support rapid ATP production, funnelling more intermediates to the TCA cycle by 48 and 72 h of temperature stress. Increased succinic acid can occur via the reversal of the second half of the TCA cycle to reduce equivalents for the synthesis of nicotinamide adenine dinucleotide (NAD^+^) and flavin adenine dinucleotide (FAD) [12]. At the remaining timepoints, a reduction of succinic acid was seen (compared to T2, which was the highest) in the temperature-stressed group, which can be attributed to the upregulation of the aspartate-succinate pathway (more succinate was thus used) to support energy production in the presence of thermal stress [35]. Notably, alpha (α)-ketoglutaric acid was the only TCA cycle intermediate in the heat-stressed group which presented lower than the control group (albeit like the controls at timepoint five). Alpha-ketoglutaric acid is a rate-determining intermediate of the TCA cycle, pivotal to energy metabolism [36], with functions in antioxidative defence, amino acid homeostasis, signalling, and genetic regulation [37]. Additionally, the role of α-ketoglutaric acid in the detoxification of reactive oxygen species (ROS) is potentially supported by other metabolites detected in the current study, such as itaconic acid, gamma-aminobutyric acid (GABA), methionine and glutathione (Figure 3), suggesting that thermal stress initiated a rapid release of ROS in the mussels at 24 °C.

### 3.3. Metabolites Countering Oxidative Stress

Oxidative stress is caused by an imbalance between the production and accumulation of ROS in cells and tissues and the inability of a biological system to detoxify them [38]. Typically, antioxidant compounds scavenge ROS or indirectly act to upregulate antioxidant defences or inhibit ROS production [39]. The production of ROS in response to thermal stress, along with an increase in antioxidant metabolites and total antioxidant capacity, is well documented for *P. canaliculus* [10]. In the current study, glutathione, an important antioxidant molecule with the ability to scavenge ROS, was detected in the highest concentration at the first sampling timepoint within the thermally stressed mussels. Possibly, temperature stress resulted in increased levels of glutathione, supporting glutathione ratios despite heat-induced oxidative stress [40]. As time progressed, the levels of glutathione decreased in the current study, indicating the utilisation of this antioxidant to counter ROS production, as seen in *P. virdis* [40]. Towards the last timepoint (T5), glutathione of the thermally stressed mussels under investigation stabilised within the ranges of the control group, possibly to support the maintenance of the cellular redox status. Reduced methionine levels detected in the heat-stressed mussels (T1-3) in the current study reflected the use of methionine for transsulfuration and within the glutathione pathway to support oxidative stress mechanisms as previously recorded in heat-stressed *P. canaliculus* [41]. Additionally, itaconic acid showed the highest increase at 72h within the current study, potentially attributing to the inhibition of mitochondrial ROS production in heat-stressed mussels [42]. Increased itaconic acid has previously been reported in *P. canaliculus* in response to thermal stress [41] and remains a metabolite of interest within mussel metabolism [43]. As was the case for glutathione, GABA was detected at the highest concentration at the start of the current experiment in mussels kept at 24 °C when compared to 16 °C. GABA accumulation controls redox homeostasis in multiple organisms by supporting antioxidant status [44]. Resultingly, the increased GABA can be ascribed to a ROS scavenging role during thermal stress. Many species have been shown to benefit from GABA’s antioxidative properties, including juvenile *Litopenaeus vannamei* [45] and juvenile Chinese mitten crab (*Eriocheir sinensis*) [46].

### 3.4. Anaerobic Energy Supply

The switch between aerobic and anaerobic metabolism is common in intertidal mussels [47], with the activation of anaerobic metabolism also previously supported in *P. canaliculus* in the presence of thermal stress [48]. Within this study, the first evidence to support anaerobic metabolism is the affected metabolite, lactic acid (Figure 3). Compared to the control group, lactic acid in the heat-stressed group was reduced at timepoint one and two, suggesting the utilisation (depletion) thereof by tissues to support high energy production [49]. Next, lactic acid production increased when the demand for ATP and oxygen exceeded supply [50], as seen at 96 h of mussels exposed to 24 °C. Likewise, increased levels of succinic acid can indicate oxidative stress in shellfish when oxygen availability is limited [51] and supports a shift towards anaerobic metabolism via the succinate pathway, as previously detected in temperature-stressed *P. canaliculus* [12]. The upregulation of carbohydrate metabolism with the production of alcohols and carbon dioxide as end products are often seen in oxygen-deprived scenarios [52], where succinic acid, ethanol, and lactic acid are produced anaerobically [53]. In the current study, both isopentyl acetate and 2-methyl-1-butanol can be considered as by-products of glycolysis, supporting energy production in anaerobic conditions, with the highest concentrations found when intermediates of the glycolysis pathway were increased (T3). Amino acids can also serve as substrates or intermediates of anaerobic energy metabolism [54], as seen by the peak of N-acetylglutamic acid (Figure 3E) at 72 h, followed by a decrease towards the end of the experiment. The transamination role of ketoglutaric acid is herein suggested, as seen in the mussel *M. edulise* (L.) when exposed to seasonal changes [55]. Upregulation of nicotinic acid (Figure 3D) and its derivatives (nicotinic acid mononucleotide and ß-nicotinic acid mononucleotide) were seen as a peak at 96 h (T4) within the stressed group of mussels. Nicotinic acid (vitamin B3) contributes to important cofactors (like NAD^+^) to regulate stress resistance and metabolism [56]. Nicotinic acid can also be converted to fumaric acid (TCA cycle) during catabolism [57], and even though fumaric acid was not a statistically significant metabolite in the current study, the role of fumaric acid in improving ATP production during thermal stress cannot be ignored [58]. Fumaric acid has been implicated as a biomarker of anaerobic metabolism in oysters (*Ostrea edulis*) [59] and clams (*Mercenaria mercenaria*) [60], underpinning the involvement of this section of the metabolism with anaerobic energy production during heat stress in *P. canaliculus*. Nicotinic acid also plays an important role in the synthesis of precursors for pyrimidine nucleotides [61], as shown in *M. coruscus* [62] and now in *P. canaliculus*.

### 3.5. Purine and Pyrimidine Metabolism

Purine and pyrimidine metabolism (Figure 3, pathways H and I) were also altered in heat-stressed *P. canaliculus* in the current study. Although no clear overall pattern can be described within the purine and pyrimidine-affected metabolites, decreases hereof can indicate the restriction of substrates involved in deoxyribonucleic acid (DNA) and ribonucleic acid (RNA) turnover and repair mechanisms, as seen in *M. galloprovincialis* exposed to pollutants [63]. Towards the end (T5-120 h) of the heat stress challenge, many purine metabolites (adenosine 5-diP, adenosine 3,5-CMP, adenylosuccinic acid, inosine, riboflavin, inosine 5-diP, 2-deoxyguanosine 5-diP) from the heat stressed mussels were decreased to the lowest point across all sampling times. Purine metabolites might have been utilised to protect organs and tissues against heat stress [64] in *P. canaliculus*. Purine changes may also lead to impaired tissue maintenance, as reported in freshwater mussels subjected to translocation stress [65]. Furthermore, the balance between energy demand and supply comes into question, as purine metabolism plays an essential role in energy supply [62], hinting at an energy deficit in the thermally stressed *P. canaliculus* following a five-day exposure to 24 °C. The involvement of the purine salvage pathway has been highlighted as an ATP replenishment mechanism in *M. galloproviancilais* subjected to thermally induced hypoxia [66], an area for further investigation in all mussel species. Increased levels of the purine metabolites, adenosine 5-monophosphate (AMP), and 2-deoxyguanosine 5-monophosphate in the heat-stressed mussels at 72 h of the experiment. These purine metabolism changes co-insides with the increases found in glycolysis and TCA cycle metabolites, emphasising the protective roles of purine metabolites to support energy production. In addition, these two purine metabolites can provide energy and delay the accumulation of NADH^+^ and H^+^, which supports glycolysis and TCA cycle functioning [67].

The pyrimidine metabolites, L-dihydroorotic acid, and uridine-5-diP, detected in the current study (Figure 3H), directly support the synthesis of uridine 5-monophosphate (UMP) and other pyrimidine nucleosides for the synthesis of DNA and RNA [68]. These pyrimidines were decreased by 120 h of heat stress, suggesting utilisation of these to produce nucleotides and subsequently increase protein synthesis in relation to elevated temperature [69]. An increase in pyrimidine bases also occurred due to heat stress, mainly after 48 h of thermal exposure. The pyrimidine metabolites, cytidine, 2-deoxyuridine, cytidine-5-monoP, and 2-deoxyxytidine 5-diP showed an increased response (at T2), possibly indicating the activation of a tolerance mechanism to protect nucleic acids and protein synthesis [70] against heat stress in *P. canaliculus*. Ultimately, exposure to a higher temperature may cause an increased requirement in thermally stressed mussels for pyrimidine metabolism due to increased RNA and DNA turnover and potential cell damage encountered [71].

### 3.6. Amino Acid Metabolism

The use of free amino acids to balance intracellular osmolality has been previously reported in *M. edulis* exposed to an array of stressors [72] and comes to light in the current investigation. For example, changes in taurine (Figure 3) levels can support osmoregulation functions [73]. Taurine concentrations were the highest at 24 h, similar to other metabolites providing antioxidant functions, supporting the use of taurine to provide antioxidant and cytoprotective roles to mussels during thermal stress, as seen in *M. galloprovincialis* [66]. Affected aromatic amino acids, tryptophan and phenylalanine (Figure 3F) showed an increase at 96 h in mussels held at 24 °C in the current study, likely due to amino acid biosynthesis in response to stress [24]. Then again, shikimic acid also showed an increase in metabolite response at 96 h of heat stress, serving as a precursor to aromatic amino acids and supporting the synthesis of proteins, vitamins, and structural blocks of electron carriers [74]. The amino acid citrulline (Figure 3, pathway E) was increased up to 48 h in the heat-stressed mussels under investigation. Citrulline increased similarly in a recent study on heat-stressed clams as a means to balance the nitrogen and ammonia ions and prevent toxicity [60]. Resultantly, the function of the urea cycle as a collection point for nitrogenous waste [75] is demonstrated where citrulline concentrations decreased towards the last few sampling points (T4 and T5) to likely support ammonia detoxification and cellular osmoregulation [76]. Increased pantothenic acid levels were seen in thermally stressed mussels in the current study, 96 h into the experiment, possibly as a method to replenish coenzyme A (CoA) levels and maintain energy production in heat-stressed mussels. Pantothenic acid (vitamin B5) is the precursor of CoA production, which is the essential cofactor of cellular metabolism involved in anabolic and catabolic reactions of lipids, carbohydrates, proteins, ethanol, bile acids, and xenobiotics [77]. Then again, amino acids also support the de novo synthesis of purine nucleotides [78].

### 3.7. Understanding Mussel Metabolite Thermal Response in an Aquaculture Context

When organisms are exposed to temperature ranges that exceed their optimum limits, the outcome is survival or death, and even if survival is the result, the fitness of the organism will potentially be affected [29]. The likelihood of surviving a marine heatwave event depends on a wide range of factors, including the integrated thermal history of the mussel [79], nutrition status, genetics, life stage, pathogen exposure, and reproductive status [80]. No studies to date (including the present study) have demonstrated significant mortality of *P. canaliculus* at 24 °C in otherwise healthy mussels [41], except when they are held at 24 °C for many months [4]. Seawater temperatures of 26 °C are detrimental to *P. canaliculus* survival, and mortality occurs after several days at this temperature. This suggests that there is a tipping point at ~26 °C where *P. canaliculus* cannot meet the physiological demands associated with this increase in temperature [80]. From a metabolic perspective, *P. canaliculus* has the potential to adapt to heat stress up to 24 °C for five days by activating costly defence and repair mechanisms, which reallocate energy away from organismal growth towards maintenance. The investigation of temperature-induced alterations at the metabolite level allows a background upon which management decisions can be founded and will support the development of tools that will further improve aquaculture techniques and mussel health. The use of metabolomics for the assessment of routine monitoring of mussel health in wild and farmed populations has applications beyond research. For example, metabolite biomarkers can help to monitor the effects of environmental stressors on mussels [81]. Considering that seasonal forecasting is currently being used to warn about marine heatwaves and inform management responses [2], proactive steps can also be taken to promote mussel health. From the current study, it is clear that mussels exposed to a marine heatwave require additional energy for organismal functioning via aerobic and anaerobic processes and upregulation of various metabolites to counter oxidative stress production. Hypothetically, these needs can be filled by ensuring adequate oxygen delivery to mussels and by supplementing their diets with amino acids. Dietary and nutritional management strategies have shown promising results in restoring the effects of thermal stress in fish [82]. Strategies of increasing aeration on mussel farms have been investigated within the context of ocean acidification and warrant future research to implement dropper aeration or integrated multi-trophic aquaculture to improve oxygen content in the face of climate stressors [83]. Open ocean aquaculture, suspending mussel lines deeper in the water column [84], and selective breeding for thermotolerance can also be used as mitigation strategies [80]. Heat hardening (the transient response that improves thermal tolerance and a plastic trait that can be modified by acclimation to different thermal regimes) can be utilised to condition mussels to environmental conditions. This strategy has enhanced mitochondrial redox potential for oxidative defence capacity and respiration in *M. galloprovincialis* [66] and remains a promising prospect for *P. canaliculus*.

## 4. Conclusions

Climate change has led to an increase in the frequency, duration, and intensity of marine heatwaves, which can cause physiological stress in mussels. These changes often involve synergistic processes and depend on the complex stoichiometric relationships of the host ecology, environmental context, and direct and indirect pathogens. While our investigation primarily aimed to assess a specific factor of heat stress associated with climate change, we acknowledge the hypothetical metabolic evidence provided regarding the complex dynamics of environmental interactions. Nevertheless, the current study presents new data concerning the potential adaptive response of *P. canaliculus* to a five-day marine heatwave exposure, along with metabolic insight on the dynamic nature of energy production in mussel haemolymph metabolites. Data from the current study may support management responses to mitigate the impacts of marine heatwaves on farmed *P. canaliculus*. In summary, mussels subjected to heat stress utilised sugar-based metabolites up to 48 h post-exposure, whereafter, a deficit of the carbohydrate sources likely promoted an increase of glucose production by 72 h to ensure ATP via substrate-level phosphorylation and the TCA cycle. Upregulation of the TCA cycle and the aspartate-succinate pathway followed by 48 h and 72 h of exposure to possibly allow rapid ATP production. The presence of oxidative stress within the thermally stressed mussels is likely at 24 h, with increased antioxidant molecules detected to scavenge ROS. However, the cellular redox status stabilised as time continued. Purine metabolism was depleted by 120 h, and pyrimidine bases were upregulated by 48 h of thermal exposure. Affected amino acids could potentially be involved in osmolality, biosynthesis, and ammonia detoxification functions. Ultimately, when marine heatwaves occur, *P. canaliculus* shows capabilities to respond and acclimate to thermal stress by regulating their energy metabolism, balancing nucleotide production, and implementing oxidative stress mechanisms over time. This study hypothesises that *P. canaliculus* has the capacity to respond to and survive short-term marine heatwave events, but their ability to rapidly acclimate to repeated events with longer timeframes remains unclear and warrants further investigation.

## Data Availability

The data presented in this study are available on request from the corresponding author. The data are not publicly available due to the value of researchers’ interaction when/if the data are requested.

## Supplementary Materials

The following supporting information can be downloaded at https://www.mdpi.com/article/10.3390/metabo13070815/s1. Table S1: Metabolomics findings of *Perna canaliculus* haemolymph metabolites with an interaction effect between temperature and time. Compounds are listed in alphabetical order with a *p*-value, a Kyoto Encyclopaedia of Genes and Genomes (KEGG) identification number, and a metabolite class grouping visible in the table.
