# Supplementary material for: Metabolite Changes of Perna canaliculus Following a Laboratory Marine Heatwave Exposure: Insights from Metabolomic Analyses
Source: Metabolites. 2023 Jul 3;13(7):815. doi: 10.3390/metabo13070815 (PMC10385441; doi:10.3390/metabo13070815)
Supplement: Supplementary file 1 [file metabolites-13-00815-s001.zip › metabolites-2437014-supplementary.pdf]

Supplementary material

Supplementary Table S1: Metabolomics findings of *Perna canaliculus* haemolymph metabolites with an interaction effect between temperature and time. Compounds are listed in alphabetical order with a p-value, a Kyoto Encyclopaedia of Genes and Genomes (KEGG) identification number and metabolite class grouping visible in the table.

**Metabolite**	**p-value**	**KEGG ID**	**Metabolite class**
2-Deoxyadenosine 5-diphosphate	4.48E-05	C00206	Purine nucleotides
2-Deoxycytidine	2.86E-03	C00881	Pyrimidine nucleosides
2-Deoxycytidine 5-diphosphate	1.73E-08	C00705	Organic oxoanionic compounds
2-Deoxy-D-ribose	1.80E-03	Not Available	Organooxygen compounds
2-Deoxyguanosine 5-diphosphate	1.40E-05	C00361	Cinnamic acids and derivatives
2-Deoxyguanosine 5-monophosphate	3.23E-04	C00362	Purine nucleotides
2-Deoxyuridine	8.08E-07	C00526	Pyrimidine nucleosides
2-Methyl-1-butanol	4.48E-05	Not Available	Organooxygen compounds
3-Methylglutaric acid	6.17E-03	Not Available	Fatty Acyls
4-Methyl-2-oxovaleric acid	2.24E-08	Not Available	Keto acids and derivatives
5-Hydroxy-3-indoleacetic acid	6.17E-03	C05635	Indoles and derivatives
Adenosine 3-5-cyclic monophosphate	4.48E-05	C00575	Purine nucleotides
Adenosine 5-diphosphate	1.73E-04	C00008	Purine nucleotides
Adenosine 5-monophosphate	1.37E-04	C00020	Purine nucleotides
Adenylosuccinic acid	3.31E-03	Not Available	Purine nucleotides
AICAR	7.38E-04	C04677	Imidazole ribonucleosides and ribonucleotides
alpha-D-Glucose-1-phosphate	1.80E-06	C00103	Organooxygen compounds
alpha-Ketoglutaric acid	7.91E-11	C00026	Keto acids and derivatives
Arabinose-5-phosphate	3.31E-03	C01112	Organooxygen compounds
beta-Nicotinamide mononucleotide	7.78E-06	C00455	Pyridine nucleotides
Cellobiose	2.23E-06	C06422	Organooxygen compounds
cis-Aconitic acid	8.47E-05	C00417	Carboxylic acids and derivatives
Cytidine	8.80E-03	C00475	Pyrimidine nucleosides
Cytidine-5-monophosphate	2.00E-04	C00055	Pyrimidine nucleotides
Cytosine	4.27E-04	C00380	Diazines
D-+-Galactosamine	2.02E-04	Not Available	Organooxygen compounds
Deoxythymidine 5-triphosphate	3.31E-03	C00459	Pyrimidine nucleotides
D-Fructose 1-6-biphosphate	4.48E-05	C05378	Organooxygen compounds
D-pantothenic acid	3.31E-03	C00864	Organooxygen compounds
D-Xylulose-5-phosphate	1.73E-04	C00231	Organooxygen compounds
gamma-Aminobutyric acid	7.78E-06	C00334	Carboxylic acids and derivatives
Glyceric acid	6.17E-03	C00258	Organooxygen compounds
Guanosine	4.48E-05	C00387	Purine nucleosides
Inosine	6.17E-03	C00294	Purine nucleosides
Inosine 5-diphosphate	8.47E-05	C00104	Purine nucleotides
Isopentyl acetate	2.23E-06	C12296	Carboxylic acids and derivatives
Itaconic acid	1.98E-08	C00490	Fatty Acyls
Lactic acid	1.54E-07	C00186	Hydroxy acids and derivatives

L-Arabinose	7.78E-06	C02479	Organooxygen compounds
L-Citrulline	6.79E-04	C00327	Carboxylic acids and derivatives
L-Dihydroorotic acid	6.20E-06	C00337	Carboxylic acids and derivatives
L-Gluthathione (oxidized)	3.80E-06	C00051	Carboxylic acids and derivatives
L-Malic acid	2.67E-03	C00149	Hydroxy acids and derivatives
L-Methionine	8.08E-07	C00073	Carboxylic acids and derivatives
L-Phenylalanine	3.31E-03	C00079	Carboxylic acids and derivatives
L-Tryptophan	3.31E-03	C00078	Indoles and derivatives
Maleic acid	1.78E-04	C01384	Carboxylic acids and derivatives
Mevalonic acid 5-phosphate	1.76E-03	C01107	Organic phosphoric acids and derivatives
m-Hydroxybenzoic acid	3.31E-03	C00587	Benzene and substituted derivatives
N-Acetylglutamic acid	4.41E-05	C00624	Carboxylic acids and derivatives
Nicotinic acid	1.18E-04	C00253	Pyridines and derivatives
Nicotinic acid mononucleotide	3.31E-03	C01185	Pyridine nucleotides
o-Hydroxy hippuric acid	5.47E-04	Not Available	Benzene and substituted derivatives
Riboflavin	1.30E-03	C00255	Pteridines and derivatives
Shikimic acid	1.73E-04	C00493	Organooxygen compounds
Succinic acid	6.08E-04	C00042	Carboxylic acids and derivatives
Succinic semialdehyde	6.92E-10	C00232	Fatty Acyls
Taurine	8.80E-03	C00245	Organic sulfonic acids and derivatives
trans-Aconitic acid	3.31E-03	C02341	Carboxylic acids and derivatives
Uridine 5-diphosphate	4.48E-05	C00015	Pyrimidine nucleotides
Xylitol	8.80E-03	C00379	Organooxygen compounds
